# The effect of eye movement desensitization and reprocessing (EMDR) on abdominal pain in patients with irritable bowel syndrome (IBS): a study protocol for a randomized controlled trial (EMDR4IBS)

**DOI:** 10.1186/s13063-023-07784-1

**Published:** 2023-12-04

**Authors:** B. Wertheim, E. E. Aarts, C. de Roos, Y. R. van Rood

**Affiliations:** 1 Diakonessenhuis Utrecht, Utrecht, The Netherlands; 2 Department of Methodology and Statistics, Utrecht University, Utrecht, The Netherlands; 3 Amsterdam UMC, Academic Center for Child and Adolescent Psychiatry Levvel, Amsterdam, The Netherlands; 4 Leiden University Medical Centre, Leiden, The Netherlands

**Keywords:** Irritable bowel syndrome, IBS, Eye movement desensitization and reprocessing, EMDR, Treatment, Abdominal pain, Quality of life, QoL, RCT, Study protocol, Linear mixed effects model

## Abstract

**Background:**

Irritable bowel syndrome (IBS) is a highly prevalent disorder for which treatment options such as medication, diets, and hypnotherapy either have shown limited effect or relieve symptoms in only a limited subset of patients. Abdominal pain is the key criterion for the diagnosis and is deemed the most distressing IBS symptom, and the most disruptive of everyday life. A growing body of research demonstrates the effect of Eye Movement Desensitization and Reprocessing (EMDR) on chronic pain. EMDR is known as a safe and successful treatment for disorders in which unresolved traumatic memories play a role in the cause or maintenance of symptoms. In IBS, activated memories may increase pain through pain flashbacks and the stress generated by unresolved memories. The aim of this study is to ascertain whether applying EMDR to traumatic memories including pain memories will reduce abdominal pain in IBS patients.

**Methods:**

This study is a randomized controlled trial which will be conducted at a city hospital in the Netherlands. Adult patients with considerable IBS pain (pain intensity at least 60/100 during at least 5/10 days) will be randomly assigned to either EMDR therapy or the wait list. We aim to include 34 participants. The EMDR condition comprises seven sessions, around 90 min in length delivered weekly, the first of which is a case conceptualization session. All participants will be assessed at baseline, post-treatment, and at 3 months follow-up. The primary outcome measure is pain intensity on a Likert scale which is self-reported daily during a 2-week period. Secondary outcomes include similar daily ratings on other IBS symptoms and reported hindrance of valued activities, and also standardized questionnaires on IBS symptoms and Quality of Life. Data will be analyzed by a Linear Mixed Effects Model for repeated measures.

**Discussion:**

The results are expected to gain insight into the effectiveness of EMDR treatment on abdominal pain in IBS. As there are very few effective treatment options for IBS-related abdominal pain, this study could have important implications for clinical practice.

**Trial registration:**

Human ethics committee MEC-U NL71740.100.20. International Clinical Trial Registry Platform: NL8894. Prospectively registered on 28 January 2020.

**Supplementary Information:**

The online version contains supplementary material available at 10.1186/s13063-023-07784-1.

## Administrative information

Note: the numbers in curly brackets in this protocol refer to SPIRIT checklist item numbers. The order of the items has been modified to group similar items (see http://www.equator-network.org/reporting-guidelines/spirit-2013-statement-defining-standard-protocol-items-for-clinical-trials/).

**Table:** 

Title {1}	The effect of Eye Movement and Desensitization and Reprocessing (EMDR) on abdominal pain in patients with Irritable Bowel Syndrome (IBS): a study protocol for a randomized controlled trial (EMDR4IBS)
Trial registration {2a and 2b}.	Human ethics committee MEC-U NL71740.100.20. Prospectively registered January 28 2020.Registered in the International Clinical Trial Registry Platform: NL8894. ICTRP Search Portal (who.int)
Protocol version {3}	Version 2.7 dated 14–12-2021
Funding {4}	This study is performed as part of the post doc training of B. Wertheim. The Diakonessenhuis Utrecht (a hospital) facilitates the study, by allowing B. Wertheim to work on the trial for 2–4 h per week. A grant was awarded by 1) the *Vereniging EMDR Nederland (*Dutch society for professionals who are registered as EMDR Europe Practitioner, or are training to be registered) (5000 euros) 2) Diakonessenhuis Science Bureau (from funds of *Stichting Vrienden van het Diakonessenhuis*) (9250 euros)
Author details {5a}	Baukje Wertheim, MSc, Diakonessenhuis Utrecht, Utrecht, The Netherlands bwerthei@diakhuis.nl Emmeke E. Aarts, PhD, Department of Methodology and Statistics, Utrecht University, Utrecht, The Netherlands e.aarts@uu.nl Carlijn de Roos, PhD, Amsterdam UMC, Academic Center for Child and Adolescent Psychiatry Levvel, Amsterdam, The Netherlands c.deroos@levvel.nl Yanda R. van Rood, PhD, Leiden University Medical Centre, Leiden, The Netherlands. y.r.van_rood@lumc.nl
Name and contact information for the trial sponsor {5b}	Investigator initiated clinical trial B. Wertheim (principal investigator) bwerthei@diakhuis.nl
Role of sponsor {5c}	This is an investigator initiated clinical trial. The funders played no role in the design of the study and will not be involved in the collection, analysis, and interpretation of data or in writing the manuscript.

## Introduction

### Background and rationale {6a}

Irritable bowel syndrome (IBS) is a common chronic disorder of brain-gut interaction. Its etiology is most likely multi-factorial involving biological, psychological, and social factors. The self-reported prevalence of IBS in the Netherlands is 15–20% in women and 5–20% in men [1]. Not only do symptoms of IBS have great impact on the individual’s quality of life [2], but it also causes a significant burden to the health care system and society at large [3]. The Rome criteria are developed and updated by an international group of experts and are used to diagnose IBS in clinical care and clinical trials. The Rome IV criteria [4] for IBS feature altered bowel habits and stool changes, but the key criterion, and a requirement for the diagnosis, is “frequent abdominal pain”.

IBS pain is often described as sharp, stabbing, cramping, or throbbing. Pain intensity may be continuous or change throughout the day, the unpredictability making it difficult to plan activities (either social or work-related).

Abdominal pain has been found to be the most distressing IBS symptom, as well as the most disruptive, having a great impact on the quality of life [5, 6]. Up to 16% of IBS patients in secondary care have contemplated suicide, because of symptom severity, i.e., pain intensity, interference with life, and lack of effective treatment [7]. Conventional pain medication is not suitable for treating IBS pain [8]. The effectiveness of drugs such as NSAIDs or opiates in alleviating IBS pain is low, and they have considerable side effects, sometimes even aggravating pain in the long run. Although other treatment options such as diets, laxatives, and antispasmodics can relieve pain in some patients, in general, patients are dissatisfied with their overall efficacy and tolerability [9]. This has led the Dutch IBS guideline for General Practitioners to center its recommendations on educating the patient, reducing anxiety and avoidance behaviors, and promoting healthy life style choices. In cases of a severely reduced quality of life, the guideline suggests either antidepressants or psychological treatment [10]. Often, patients prefer the last option. Of these psychological treatments for IBS, so far, hypnotherapy appears to be most effective in pain reduction [11–16]. However, a review shows that response rates found in different studies vary widely [14]. Hence, the search for effective treatments of abdominal pain in IBS continues.

In the last decades, there has been a growing body of research on the effect of eye movement desensitization and reprocessing (EMDR) on chronic pain. EMDR is known as a successful and widely embraced treatment for post-traumatic stress disorder (PTSD) and other disorders in which unresolved traumatic memories play a role in the cause or maintenance of symptoms [17–19]. Four systematic reviews, published in 2009, 2014, 2016, and 2019 [20–23], conclude that EMDR is an effective treatment for chronic pain. Clinical practice and several published case studies established that traumatic experiences involving physical pain can cause pain flashbacks. These pain flashbacks are usually very similar to the original pain in terms of location and quality [24–27] and can be triggered by both internal and external trauma-related cues. Activated pain memories can maintain current pain through pain flashbacks and the stress generated by unresolved memories [28].

Research into the pathogenesis of abdominal pain in IBS appears to confirm this notion. In IBS, neurobiological research has shown altered processing of signals along the gut-brain axis. These changes appear to be related to pain-related expectations and learning processes [29]. It is suggested that when interoceptive memories of aversive visceral states develop, they may allow the brain to recall visceral experiences of pain in the form of constant or recurrent pain [30].

A successful EMDR treatment in PTSD helps patients (re)process traumatic memories, thereby reducing memory vividness, emotionality, and hypervigilance. In (re)processing the traumatic pain memories, we expect to reduce their chronic (re)activation, optimizing the circumstances for recovery and thereby reducing pain intensity. We designed this study to ascertain whether applying EMDR to traumatic memories including pain-related memories will reduce abdominal pain in IBS patients. Although clinical practice shows promising results, to our knowledge, no research into the effect of EMDR on abdominal pain in IBS has been conducted.

## Objectives {7}

The primary objective of this study is to assess the effect of EMDR treatment on abdominal pain in IBS. It is hypothesized that participants in the EMDR treatment condition will show a greater reduction in abdominal pain intensity than those in the wait-list control group.

Our secondary objectives are to determine the effects of EMDR treatment on (1) other salient IBS symptoms (personalized), (2) overall severity of IBS-related symptoms, (3) the hindrance of valued activities caused by abdominal pain (personalized), (4) quality of life, and (5) the reported rate of adequate relief from IBS symptoms.

It is hypothesized that EMDR treatment will, as compared to the wait-list control condition, lead to a significantly greater reduction of salient symptoms, the overall severity of IBS symptoms, and the experienced hindrance of valued activities. Furthermore, we hypothesize that quality of life will show greater improvement in the treatment condition (EMDR) than in the wait-list control condition, and that after treatment and at follow-up, a higher percentage of patients in the treatment condition (EMDR) will indicate experiencing “adequate relief” of IBS symptoms than in the wait-list control condition.

## Trial design {8}

The present study is a superiority study comparing the EMDR intervention with a wait-list control group in a two-armed randomized controlled trial. Patients will be randomly allocated to either EMDR treatment or wait list, with an equal allocation ratio. Blinding for the intervention is not possible.

This study is registered at The Netherlands Trial Register, with registration number NL8894. It is thereby automatically included in the International Clinical Trial Registry Platform.

**Fig. 1:**
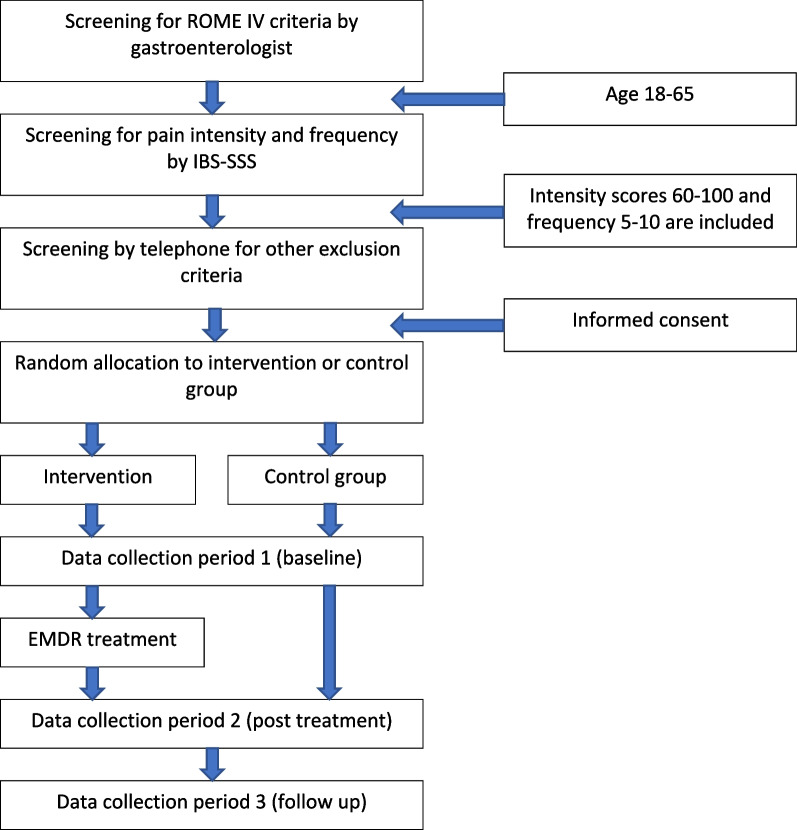
Inclusion flowchart

For an overview of the study design, including recruitment, random allocation, assessments, and treatment, see Fig. 1.

## Methods: participants, interventions, and outcomes

### Study setting {9}

Participants will be recruited from the general hospital Diakonessenhuis, Utrecht area in the Netherlands.

### Eligibility criteria {10}

Detailed inclusion and exclusion criteria are listed in Table 1.

**Table 1:** Detailed inclusion and exclusion criteria

Inclusion criteria	
1	Age 18–65
2	Meet Rome IV diagnostic criteria for IBS
3	A pain intensity score of at least 60 (on a scale of 0–100)
4	Frequency of abdominal pain at least 5 out of 10 days
5	Informed consent
Exclusion criteria	
1	Insufficient proficiency in the Dutch language, or any other circumstances interfering with communication or completing questionnaires
2	Psychiatric problems such as psychosis, severe depression, or suicidality requiring immediate treatment
3	Ongoing trauma-focused treatment, such as EMDR, trauma-focused Cognitive Behavioral Therapy (tCBT), and Imaginary Exposure (IE)
4	Any other medical conditions such as Colitis or Crohn’s disease in which abdominal pain is a key symptom
5	Pain in another area of the body which is more prominent than abdominal pain
6	Ongoing drug abuse (excluding nicotine or caffeine abuse) interfering with EMDR therapy
7	Self-reported pregnancy or planned pregnancy within the next 5 months. Sensations in the abdomen caused by the pregnancy might be difficult to distinguish from the sensations caused by IBS

Subjects are eligible for participation if they meet Rome IV criteria for IBS and report severe pain intensity at least half of the time. Severity of pain is measured by the self-reported pain intensity score on the Irritable Bowel Syndrome—Severity Scoring System (IBS-SSS). A pain intensity score of 60 or more on a scale of 0–100 is considered to reflect “severe pain.”

The Rome IV criteria [4] are:

Recurrent abdominal pain, on average, at least 1 day/week in the last 3 months, associated with two or more of the following:

- Related to defecation
- Associated with a change in frequency of stool
- Associated with a change in form (appearance) of stool.

Criteria need to be fulfilled for the last 3 months with symptom onset at least 6 months prior to diagnosis.

Participants are allowed to continue any prior IBS treatment such as medication or a diet as long as they agree not to change these during the trial.

Treatment will be administered by qualified psychologists who have been included in the Dutch legal register for Professions in Individual Health Care (BIG-register) and are at least EMDR Europe accredited level 1 trained in EMDR therapy.

### Who will take informed consent? {26a}

The gastroenterologist establishes whether subjects meet Rome IV criteria for IBS. Afterwards, they complete a short questionnaire comprised of the IBS-SSS (short form) to which this question is added: May we invite you to participate in a scientific study of a new treatment for IBS?

Subjects meeting the initial criteria (pain intensity and frequency) receive, by post, the Patient Information Form packet (PIF) approved by the Medical Ethical Committee. About a week later the principal investigator calls the subject to answer questions that may have arisen and to screen for exclusion criteria. If the subject meets inclusion criteria, does not meet exclusion criteria, and is willing to participate, he or she fills out and signs the consent form included in the PIF and sends it to the principal investigator in the included return envelope.

### Additional consent provisions for collection and use of participant data and biological specimens {26b}

Standard items in the consent form state that the participant agrees that

- he or she has been informed satisfactorily about the study and that enough time was given to form a well-founded opinion on participation.
- he or she has chosen to participate voluntarily, and he or she is free to stop participation without having to give a reason.
- she knows she cannot participate when pregnant.

and agrees to

- the principal investigator informing both their gastroenterologist and general practitioner of their participation in the study and of any unexpected findings concerning their health.
- the collection of data in order to answer the study’s research questions.
- allow the study process controllers specified in PIF, access to their data.
- the therapy sessions being videotaped to check for treatment adherence and fidelity.

Additionally, the participant is asked to state whether they consent to

- being recognizable in de session video tapes.
- their personal information and data being used in future studies on either EMDR or IBS.
- being approached, after this study, to participate in follow-up research.

### Interventions

#### Explanation for the choice of comparators {6b}

So far, no scientific research has been done to study the effect of EMDR on pain in IBS. We choose to compare the intervention group with a wait-list control group. Comparison to an active control group such as gut-directed hypnotherapy or psychoeducation would require a larger sample size than we have the means to carry out. The same restrictions in time and resources prevent us from arranging an inactive control condition.

#### Intervention description {11a}

EMDR treatment is based on the eight phases of the EMDR standard protocol [31–33]. Listed below are the principal steps in each phase.

1. History taking: taking history, making a case conceptualization based on the information gathered, and compiling a list of traumatic experiences.
2. Client preparation: explanation of the theory behind EMDR, the introduction of the eye-movement task and tactile and auditory tasks (buzzers, clicks), answering questions, in general preparing the client for EMDR.
3. Assessment: the memory that will be processed in the session is decided on. The target image (the most distressing image of the memory) is identified, as well as the principal negative belief, feelings, and sensations linked to the memory. Also, a positive cognition is chosen. Baseline measures are established for the credibility of this positive cognition (Validity of Cognition, VoC), and the amount of stress generated by the target (Subjective Units of Distress, SUD).
4. Desensitization: taxing working memory while focusing on the target, by applying eye movements, until the SUD score is zero.
5. Installation: strengthening of the positive belief until it feels true.
6. Body scan: the therapist asks the client to bring the original target to mind and check for any residual tension in their body. If so, these physical sensations are then targeted for reprocessing.
7. Closure: consolidation of the positive effects (whether the reprocessing is complete or not), instructions for between sessions and how to reach the therapist when needed.
8. Reevaluation of treatment effect: takes place at the beginning of each new session, to check whether the SUD of previous targets is still low.

After that another memory from the trauma list is chosen, for which the procedure is repeated from phase 3.

A special kind of target is the flash forward, which does not involve a memory, but an image representing a dreaded event in the future. Interweaves can be used when the reprocessing process is blocked or loops. Extra tasks taxing working memory can be used in case of high-stress levels of abreaction.

In this study, the intervention consists of seven weekly 90-min sessions, which include:

- An intake in which onset, severity, frequency, and impact of IBS symptoms are explored and an overview is made of all the traumatic events related to the onset and/or maintenance of the abdominal pain or negative experiences with the pain. Both PTSD A criterion events such as rape or car accident and adverse events that are not life-threatening such as bouts of severe abdominal pain or instances of (near) defecation in public are included in the overview. Based on the acquired information, a case conceptualization is made, from which the memories to be processed are selected. This concludes phases 1 and 2 of the EMDR standard protocol.
- Six sessions of EMDR lasting 90 min each in which phases 3 to 8 of the EMDR standard protocol [31–33] are applied to the selected memories. In addition to the customary sets of rapid eye movements, tactile and auditory input (buzzers and beeps) may be applied to optimally tax working memory. During the EMDR sessions, the therapist will direct extra focus/attention to physical sensations, usually abdominal sensations. If relevant, a flash forward will be desensitized. Interweaves will be applied if necessary. Successful desensitization is defined as the reaching of SUD = 0 (Subjective Units of Distress; the recollection of the event no longer causes the subject any distress). If severe abdominal pain remains after having processed all relevant targets, the pain itself will become the focus of the next EMDR session. Treatment is ended either after six 90-min sessions, or when all relevant memories have been successfully desensitized.

Treatment takes place face-to-face. However, if necessary due to quarantine during the COVID-19 pandemic, it can also be offered online via video consultation. Although as yet little is known about the effectiveness of online EMDR treatment for PTSD, so far results are promising that effects may be similar to real-life EMDR treatment [34].

#### Criteria for discontinuing or modifying allocated interventions {11b}

With acquiring new information during sessions, the case conceptualization can change a little, which in turn can change the selection of memories to be processed. The EMDR treatment in itself will not be altered.

Patients are free to withdraw from partaking in the study at any time. The research team can decide to remove a patient from the study if.

1. There are serious medical reasons.
2. The subject fails to complete the questionnaire(s) during the first period of measurements.
3. The subject fails to appear at a treatment session more than twice (without notice).
4. The subject’s non-cooperation seriously hinders treatment.
5. Subject meets an exclusion criterion along the way (e.g., becomes pregnant or an intestinal disease is diagnosed).
6. There are persistent serious side effects.

Like all studies involving human subjects, we are required to report serious adverse events as defined by the Central Committee on Research Involving Human Subjects (in Dutch: CCMO, Centrale Commissie Mensgebonden Onderzoek).

No serious adverse effects have been found in studies of EMDR in diverse populations. Some discomfort—both physical and psychological—both during sessions and in the first 4 days after an EMDR session is not unusual. As this is part of the psychological process of processing negative memories, this is no cause for discontinuing the intervention.

Any unexpected adverse effects will be discussed within the research team, which includes two EMDR Europe registered EMDR consultants with extended expertise. Depending on the outcome of this deliberation, steps will be taken to reduce the adverse effects. If necessary, participation in the study will be temporarily interrupted. If the adverse effects persist, participation in the study will be discontinued.

#### Strategies to improve adherence to interventions {11c}

Prior to the inclusion of a participant, an explanation about EMDR therapy is given in the Patient Information Form and, if desired, questions are answered during the telephone call in which exclusion criteria are checked. Again, later, during the intake subjects are informed thoroughly about what to expect during and after an EMDR treatment session. This increases motivation and adherence to the therapy once treatment has started.

Beforehand, trial therapists are required to demonstrate their competence in applying the treatment. As yet, there are three trial therapists, but more may be added later. To ensure treatment quality and adherence to the EMDR standard protocol, the therapists receive monthly supervision sessions by the EMDR consultants based on the EMDR treatment sessions videotaped during the study. Furthermore, weekly session checklists are viewed by the consultants to closely follow the EMDR process. Any questions by the therapists will be answered before the next session. Also, every session is videotaped to enable regular fidelity checks. Fifteen percent of all recorded sessions will be reviewed for treatment fidelity using the Treatment Integrity Checklist for EMDR. Fidelity checks will be done at random, by the last author (YvR), who is an EMDR Europe registered EMDR consultant with extended expertise.

#### Relevant concomitant care permitted or prohibited during the trial {11d}

Concurrent treatment for psychotrauma is an exclusion criterion. Upon entering the study, participants agree to refrain from starting other treatments (medication, nutritional supplements) or behavioral changes (e.g., diet) aimed at the IBS symptoms.

Treatments and diets that are already part of the patients’ customary regimen can be continued. We ask subjects to report any changes in their prescribed medication. At three measurement moments, subjects are explicitly asked about their current medication use.

#### Provisions for post-trial care {30}

Subjects from both the intervention and wait-list control group will after the end of the study meet with the principal investigator to determine if there is (still) a wish or need for treatment for the IBS symptoms. If so, they are offered treatment appropriate to their current situation, provided that this can be offered by the Department of Clinical Psychology of the Diakonessenhuis. In some cases, referral to mental healthcare may be the best course of action.

No harm is expected to occur from participating in the study and the medical ethical committee has agreed to grant an exemption for an insurance for the participants of the study. The hospital, however, has, as required by law, a liability insurance for compensation of those who suffer from harm incurred in the hospital.

### Outcomes {12}

The primary outcome of this study is the intensity of abdominal pain. As the intensity of abdominal pain in IBS can vary greatly from day to day, we have chosen to pose the same question each day for 2 weeks. Participants answer the question: “How much did you suffer from abdominal pain during the last 24 h?”. The subject’s answer is given on a 21-point Likert scale (0 = no pain, 10 = worst pain imaginable, half points possible).

In addition to the primary outcome measure (abdominal pain), we pose four other diary questions at the same time, during the same period, with the same 21-point Likert scale. Based on their statements at inclusion about their principal IBS symptoms, we ask about their top two symptoms (abdominal pain excluded): “How much did you suffer from (complaint) during the last 24 h?” (0 = not at all, 10 = extreme suffering, half points are possible). These symptoms could be frequently occurring IBS symptoms such as diarrhea, constipation, and bloating, but they can also be more idiosyncratic such as dizziness. We also ask about the hindrance subjects have experienced from their IBS symptoms when engaging in everyday activities. Based on their statements at inclusion about the two- valued activities that are most impeded by the IBS symptoms, we ask for every one of these activities: “How much hindrance have you experienced from your IBS symptoms when engaging in this activity?” (0 = no hindrance at all, 10 = made completely impossible; half points possible). Activities most likely to be impeded by IBS symptoms are working, doing sports, going out for dinner, and going out (dancing, theatre, etc.).

The 14-day-sequence of these 5 “diary questions” is administered at inclusion, 8 weeks after inclusion (for the intervention condition this means after treatment), and at follow-up (20 weeks after inclusion, 3 months after treatment).

In addition to the diary questions, we administer several questionnaires, the purpose of which is to describe the population and to either confirm or disconfirm the results of the diary questions. They also enable comparing outcomes with those of other studies. The questionnaires are administered on the last day of every 14-day-sequence of diary questions.

The questionnaires administered are:

1. IBS-SSS (Irritable Bowel Syndrome—Severity Scoring System) [35].

The first part of this questionnaire consists of 5 questions regarding the frequency and intensity of pain, the intensity of bloating, satisfaction with bowel movements, and the impact of IBS on the subject’s life in general. The answer type varies per question (Visual Analog Scale 1–100 or a specific number). Scores can range from 0 to 500 with higher scores indicating more severe symptoms (75–175 mild, 175–300 moderate, > 300 severe). The IBS-SSS severity score is recognized by the Rome Foundation as an appropriate measure for assessing changes in research on treatments [36]. A decrease of 50 points is considered a clinically relevant improvement.

Part two of the IBS-SSS consists of 5 questions that explore the nature and quality of the symptoms. In this study, only the localization of abdominal pain is addressed.

2) IBS-QOL (Irritable Bowel Syndrome—Quality of Life measurement) [37]

This questionnaire measures the impact of IBS on quality of life. It contains 34 statements covering the following 8 areas: Dysphoria, Interference with Activity, Body Image, Health Worry, Food Avoidance, Social Reaction, Sexual, and Relationships. Subjects are asked to indicate to what extent each of these statements applies to them on a 5-point Likert scale (1 = not at all, 5 = very much). The individual responses to the 34 items are summed and averaged for a total score and then transformed to a 0–100 scale for ease of interpretation with higher scores indicating better IBS-specific quality of life. In addition to the total score, a scale score can be calculated for each of the 8 areas mentioned. Distribution of the IBS-QOL is managed by the Rome Foundation. Psychometric properties are well established [38].

3) AR (Adequate Relief Question)

This is one question: “In the last 7 days, have you had adequate relief of your IBS symptoms?” The answer can be yes or no. The Rome Foundation accepts and recommends this outcome measure in RCTs [39, 40]. In this study, AR is included as an outcome measure to allow comparison of our results to those of studies using this recommended outcome measure.

Other questionnaires, not used as outcome measures, but solely administered to describe the population are:

- LEC-5 (Life Events Checklist for the DSM-5) [41]

This questionnaire is a self-report measure designed to screen for potentially traumatic events in a respondent's lifetime. It describes 17 (A criterion) events, and subjects are asked to indicate whether they have experienced such an event and in what way (happened to me, witnessed it, learned about it, part of my job, not sure, doesn’t apply). There is no formal scoring protocol or interpretation per se, other than identifying whether a person has experienced one or more of the events listed. The LEC-5 in combination with the PCL-5 can be used to screen for PTSD.

In this study, the LEC-5 in combination with the PCL-5 is used to screen for PTSD criteria.

b) PCL-5 (PTSD Checklist for the DSM-5)

This self-report questionnaire consists of 20 items concerning the 20 DSM-5 symptoms of PTSD. For each symptom, subjects are asked to indicate on a Likert scale to what extent they have suffered from it in the past month (0 = not at all, 1 = a little bit, 2 = moderately, 3 = quite a bit, 4 = extremely). A total symptom severity score (range 0–80) can be obtained by adding the scores of the 20 items together. DSM-5 severity scores for the individual symptom clusters can be obtained by summing the scores of the items of a particular cluster. The PCL-5 can be used in combination with the LEC-5 (see below) as a screening tool for PTSD. An indication for a PTSD diagnosis can be obtained by counting any item with a score of 2 (moderate) or higher as a symptom that is present. Then the DSM-5 diagnostic calculation rule is followed to ascertain whether the required criteria are met: a minimum of 1 B-symptom (questions 1–5), 1 C-symptom (questions 6–7), 2 D-symptoms (questions 8–14), and 2 E-symptoms (questions 15–20). The PCL-5 can also be used to determine the course of PTSD symptoms. Results from the USA with the previous version of the PCL (PCL for DSM-IV) suggest that a 5–10-point change indicates a reliable change and a 10–20-point change indicates a clinically significant change [42].

In this study, the PCL-5 is used to assess the severity of PTSD symptoms.

Diary questions and questionnaires are administered digitally, using an ISO 9001-certified online tool. Subjects receive an e-mail containing a link to the diary questions or the questionnaire, which they then can fill out.

### Participant timeline {13}

The recommended schematic diagram is showed in Additional file 1.

**Fig. 2:**
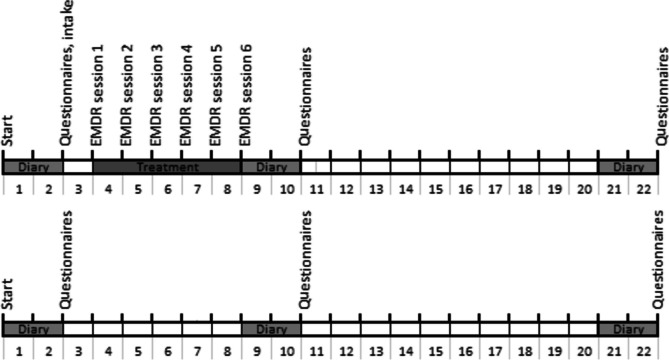
Participant timeline overview for both EMDR treatment group and wait-list control

*The intake session takes place the day after the questionnaires, and the other sessions take place on the same day, each a week later. See Fig. 2 for the overview.

**The questionnaires are administered on the last day of the 2-week diary period.

### Sample size {14}

Several factors have influenced the sample size in this study: setting, method of analysis, and the estimate of comparability with previous scientific research.

As the study is conducted as part of the curriculum of a training course taken by the principal investigator, both time and resources are limited. Our aim is to demonstrate a clinically relevant treatment effect regarding the primary outcome measure with a limited sample size.

The design of our study enables us to analyze our primary outcome data (pain score diary) using a linear mixed effects models analysis. Sample size was computed using the online statistical calculation program GLIMMPSE (General Linear Mixed Model Power and Sample Size): https://glimmpse.samplesizeshop.org/#/ using an alpha of 0.05 and a desired minimum power of 0.80.

The most important estimated value required by the GLIMMPSE program is the expected effect size. Since a study on the effect of EMDR on abdominal pain in IBS has never been done before, a search was made for comparable research. A review on EMDR treatment for chronic pain found large effect sizes [22]. The EMDR for phantom limb pain study design is most comparable to our study design [28]. Based on this study, a remarkably large effect size d of 1.22 was calculated. On entering this effect size, GLIMMPSE calculated that a sample size of 12 should be sufficient. However, when a demonstration of an average effect size is wanted, a sample size of 24 is needed. This sample size is adequate to analyze the primary outcome measure daily pain scores using a linear mixed effects model. In addition, this sample size is also expected to be adequate to analyze the secondary outcome measures from the standardized questionnaires (measures taken only three times in total) with LME models. Taking drop-out into account we decided to include a maximum of 34 participants.

### Recruitment {15}

An agreement has been made with all gastroenterologists and physician assistants in gastroenterology at Diakonessenhuis that—at the end of their consultation—they will issue the initial screening questionnaire to all their outpatients who meet the Rome IV criteria for IBS. This initial screening instrument, which encompasses the first 5 questions of the IBS-SSS, is also used as a routine outcome measurement in gastroenterology for more general purposes. The time invested by the gastroenterologist at the outpatient GI clinic is kept at a minimum, and regular reminders to hand out the screeners are sent to all gastroenterologists.

An initial estimate was made by the liaison gastroenterologist of how many patients are expected to meet the initial criteria. This estimate turned out to be too optimistic; in the first 6 months, about 30 screening questionnaires were filled out. Roughly 25% of these patients (*N* = 7) met the initial inclusion criteria.

## Assignment of interventions: allocation

### Sequence generation {16a}

The allocation sequence is generated in advance by using the Excel randomization function. Allocation of treatment vs wait list (control) is 1:1.

### Concealment mechanism {16b}

The outcome of the randomization is placed in 34 opaque, sealed envelopes, and the rank number of inclusion is written on the envelope. This is done by an independent person, not involved in the study. The sealed envelopes are kept in a locked cabinet. After receiving informed consent, the participant is assigned an ID number, after which the corresponding envelope is opened. Once allocated to either treatment or wait-list condition, blinding is no longer possible.

### Implementation {16c}

An independent person generates the allocation sequence and prepares and keeps the sealed envelopes. The main investigator will obtain the next sealed envelope from this person after informed consent was given. The note of allocation (treatment or control) and the numbered envelope are attached to the informed consent form. The main investigator then informs the participant about allocation. Allocation to a specific therapist depends mostly on the therapists’ schedules and timing of enrollment.

## Assignment of interventions: blinding

### Who will be blinded {17a}

Blinding to treatment is impossible for both participants and therapists. Treatment allocation is not discussed with the research assistant who gathers the data from the online tool (Exploratio), unless circumstances demand it. For example, the timing of questionnaires has to be changed when a treatment session was missed. The statistician engaged to perform the analysis is blinded to treatment allocation.

### Procedure for unblinding if needed {17b}

Not applicable, because blinding to treatment is impossible in the first place.

## Data collection and management

### Plans for assessment and collection of outcomes {18a}

All outcome measures are self-report questionnaires (see Outcomes {12}), which are administered via an online tool. The coded data will be extracted from the tool and saved “as is” to the secure research archive.

The diary questions and questionnaires used are described in the Outcomes section {12}.

More information on the questionnaires administered can be found in Table 2.

**Table 2:** Information on the questionnaires

IBS-SSS	Francis, C. Y., Morris, J., & Whorwell, P. J. (1997). The irritable bowel severity scoring system: a simple method of monitoring irritable bowel syndrome and its progress. Aliment Pharmacol Ther, 11(2), 395-402. 10.1046/j.1365-2036.1997.142318000.x *Dutch translation by C. Flik en E. de Winter*
IBS-QOL	Patrick, D. L., Drossman, D. A., Frederick, I. O., DiCesare, J., & Puder, K. L. (1998). Quality of life in persons with irritable bowel syndrome: development and validation of a new measure. Dig Dis Sci, 43(2), 400-411. 10.1023/a:1018831127942
ARQ	Mangel, A. W., Hahn, B. A., Heath, A. T., Northcutt, A. R., Kong, S., Dukes, G. E., & McSorley, D. (1998). Adequate relief as an endpoint in clinical trials in irritable bowel syndrome. J Int Med Res, 26(2), 76-81. 10.1177/030006059802600203 *Dutch translation by C. Flik en E. de Winter *
LEC-5	Life Events Checklist for DSM-5 © *International version*: Weathers, Litz, Keane, Palmieri, Marx, & Schnurr – National Center for PTSD (2013). © *Dutch translation by Boeschoten, M.A., Bakker, A., Jongedijk, R.A. & Olff, M. (2014)*
PCL-5	Blevins, C. A., Weathers, F. W., Davis, M. T., Witte, T. K., & Domino, J. L. (2015). The Posttraumatic Stress Disorder Checklist for *DSM-5* (PCL-5): Development and initial psychometric evaluation. *Journal of Traumatic Stress, 28*, 489-498. 10.1002/jts.22059 PTSD-Checklist for the DSM 5 © *International version*: Weathers, Litz, Keane, Palmieri, Marx, & Schnurr – National Center for PTSD (2013) © *Dutch translation by Boeschoten, M.A., Bakker, A., Jongedijk, R.A. & Olff, M. (2014)*

### Plans to promote participant retention and complete follow-up {18b}

In the first conversation on the telephone, the principal investigator establishes a positive atmosphere, in which the candidate feels free to ask questions and discuss any misgivings they might have. After the candidate has decided to participate, the investigator relates the particulars about data collection. Content (diary questions), timing, and the limited time required to fill out the questions/questionnaires of assessment are discussed. Participants are also informed that they will receive a reminder (by telephone call) from the research assistant when the diary questions or questionnaires are not filled out by a specific time in the evening. This specific time is chosen in agreement with the participant. The investigator notifies the participant that an evaluation interview will take place after data collection, in which study participation will be reviewed, and participant’s wishes concerning further treatment will be discussed. Also, participants are informed that they will receive a compensation of €25 when all questions and questionnaires have been filled out, and that participants in the treatment condition will receive a contribution for their travel costs of €6 for each visit.

The principal investigator encourages the participant to contact her with any questions or difficulties they might have filling out the diary questions or questionnaires.

When a participant has forgotten to answer the diary questions, or to fill out the questionnaires, the research assistant calls them and kindly reminds them of the e-mail, and asks them to fill out the questionnaire. A friendly atmosphere in these calls is essential.

### Data management {19}

Each participant will be allotted a unique ID number. The data from the diary questions and questionnaires are gathered using a secure connection and are stored in an online, password-protected, secured database that is only accessible by the researchers. The data are extracted and saved to the research archive coded with the unique identification number, first in their original format (separately for each participant and each day), and after that data are imported to the overall data collection file in Excel. The original format files serve as a backup. The data will be monitored for consistency and validity (e.g., check for errors, range checks, missing values) at collection by the research assistant and later by the principal investigator. The overall data file will be exported to the statistical analysis program.

Any other data will be stored under the identification number only. The coding list with identification numbers and names and the informed consent forms will be kept separate and secured by the principal investigator.

Data from diary questions, questionnaires, and treatment sessions’ video footage will be kept for 15 years, and patient files (intervention group) will be kept for 20 years in accordance with national law. Modifications to this protocol will be submitted to the approving ethical committee and the institutional review board.

### Confidentiality {27}

See {19}.

### Plans for collection, laboratory evaluation, and storage of biological specimens for genetic or molecular analysis in this trial/future use {33}

No biological specimens are gathered in this study.

## Statistical methods

### Statistical methods for primary and secondary outcomes {20a}

The statistical analyses will be performed according to the intention-to-treat principle.

The primary outcome measure in this study is the reported intensity of abdominal pain, which is registered during 14-day periods of daily diary questions, at three points in time, yielding 42 data points per participant.

The principal research question will be answered by performing a linear mixed effect models analysis, based on 2 groups (treatment and control) and 3 (baseline, after treatment, follow-up) × 14 (days) measuring moments. Before the analysis, data will be checked for outliers and the assumption of linear relations. Values with an absolute *z*-score > 4 will be treated as outliers and removed.

The model will be built in a stepwise model, first fitting an intercept-only linear mixed effects model, allowing to disentangle variance between patients and variance within patients over time. In a second step, the predictor time will be added, to control for any unexplained changes over time in subsequent models. If necessary, a quadratic term for time will be added to the model as well. Subsequently, in the third model, the fixed effect for condition will be added. In the fourth model, the fixed effect for measurement period (baseline, treatment, and follow-up) will be added (note, this variable will represent the difference between baseline and treatment period and baseline and follow-up period irrespective of condition). In the sixth model, the difference between measurement periods will be allowed to vary over patients (a so-called random slope for measurement period). If this is significant, in a seventh model, an interaction between measurement period and condition will be included, to explain why the differences between periods vary over patients. This last term, the interaction between measurement period and condition, answers our primary research question: what is the overall difference in changes in pain score over measurement periods between condition and control group?

After performing the analysis, the assumption of normally distributed errors for both level 1 and level 2 errors will be assessed.

As to the secondary outcome measures: the data gathered from the residual diary questions (pertaining to other IBS symptoms and hindrances experienced in valued activities) are analyzed in the same way as the primary outcome (abdominal pain intensity).

The outcome measures from the two standardized questionnaires, which only yield 3 data points, will be analyzed by LME models based on 2 groups (treatment and control) and 3 measuring moments (at baseline, after treatment, and at follow-up). See Fig. 2.

For all analyses regarding symptom improvement, a one-sided significance threshold of alpha = 0.05 will be used. For all other analyses, a two-sided* p* value of 0.05 will be used as the significance threshold. We will report both *p*-values and 95% confidence intervals.

Based on the Adequate Relief Question, and based on the presence of a clinically relevant reduction of symptoms as measured by the IBS-SSS, a Number Needed to treat will be calculated, for two measuring moments: after treatment and at follow-up.

### Interim analyses {21b}

No interim analyses or other stopping guidelines will be applied.

### Methods for additional analyses (e.g., subgroup analyses) {20b}

If the collected sample size allows it, it will be investigated whether observed differences between patients in treatment effect can be explained by other variables such as sex, age, symptom severity at baseline, the severity of PTSD-related symptoms at baseline, and the number of other ongoing treatments (such as diets, etc.).

In describing the study population, the following data will be reported:

- The frequency of reported IBS symptoms and valued activities (as reported at initial screening).
- The percentage of patients that meet the criteria for PTSD (as determined by LEC-5 and PCL-5 results).
- Average and standard deviation scores on PCL-5, per group and per measuring moment.
- The number of patients that have experienced additional traumatic experiences during the time of participation.

Chi-square tests or *t*-tests will be done to test for a priori differences between the intervention and control group regarding demographic and descriptive values. Both *p* values and standardized differences will be reported.

### Methods in analysis to handle protocol non-adherence and any statistical methods to handle missing data {20c}

Every effort is made to avoid missing data. The way the diary questions and questionnaires are administered online makes it impossible to skip a question. Also, patients who do not complete a questionnaire or diary will receive one or more reminders.

For all analyses, a linear mixed effects model (LMEm) analysis is used. The advantage of linear mixed effects modeling is that the analysis can be performed on all data available, and missing data is handled automatically assuming Missing at Random (MAR) without requiring imputations.

### Plans to give access to the full protocol, participant-level data, and statistical code {31c}

Statistical code will be made available through a publication via https://zenodo.org/.

See {31a} for further information.

## Oversight and monitoring

### Composition of the coordinating center and trial steering committee {5d}

As this is a small mono-center study. The study board which acts as a steering committee consists of only two people, the principal investigator and the primary EMDR consultant. The study board meets every month and reviews the progress of the study. Final decisions on changes to the protocol, publications, and reporting will be made by the study board.

The principal investigator keeps in regular contact with the gastroenterologists, research assistant, and trial therapists and provides them with day-to-day support.

This study will not be audited by independent auditors. There is also no data monitoring committee as the study involves minimal risk. The intervention (EMDR) is part of regular care.

### Composition of the data monitoring committee, its role and reporting structure {21a}

See {5d}.

### Adverse event reporting and harms {22}

The research protocol, including plans for managing adverse events, has been reviewed and approved by the human ethics and research committee MEC-U (reference number: NL71740.100.20).

As is usual when applying psychological interventions, patients may temporarily experience mild reactions such as fatigue and heightened emotionality, which is seen as the result of the ongoing effect of the intervention.

Any serious adverse effects (SAEs) as defined by the Central Committee on Research Involving Human Subjects (CCMO in Dutch) will be documented and reported immediately to the principal investigator. It will be determined whether the SAE is related to any trial procedure or intervention.

### Frequency and plans for auditing trial conduct {23}

The study will not be audited by independent auditors. To this, the human ethics and research committee MEC-U has given its assent.

### Plans for communicating important protocol amendments to relevant parties (e.g., trial participants, ethical committees) {25}

Any protocol modifications which may affect the conduct of the study or patient safety require formal amendments to the protocol. These will be agreed to by the study board and must be approved by the MEC-U ethics committee and the institutional review board prior to implementation. The results of amendments will be communicated to trial participants if relevant.

Minor changes to the protocol (e.g., corrections, clarifications that have no effect on the way the study is conducted) will be agreed upon by the study board.

## Dissemination plans {31a}

After completion of the study, the results will be submitted for publication to a peer-reviewed scientific journal, regardless of the direction or magnitude of effects. Also, we intend to present the results at relevant research conferences, and in (inter)national publications. And finally, if results warrant it, they will be used for educational and training purposes.

The institutions providing grants will not impose restrictions on publication.

Participants will receive the final trial report on request, as well as any other parties who have shown an interest (such as the participants’ general practitioners). Also, participants will be offered an overview of their own results on several outcome measures.

The research protocol, data, and statistical code supporting the findings of the final trial report will be available on request, if deemed reasonable and in accordance with the EU general data protection regulation.

The investigators involved and the statistician engaged for the analysis will be named as (co-) authors of the publication of the final trial report.

## Discussion

This article described the study protocol of a hospital-based RCT on EMDR and wait-list control in IBS patients. The primary aim of the study is to gain insight into the effect of EMDR treatment on abdominal pain in patients with IBS. As IBS is a highly prevalent condition, and there are very few effective and acceptable treatment options for IBS in general, and for IBS-related abdominal pain specifically, this study could have important implications for clinical practice. If we find that EMDR is indeed effective in reducing abdominal pain in IBS, this study may contribute to important advances in the psychological treatment of patients with IBS and thereby might have considerable positive impact on both the individual lives of IBS sufferers and the societal burden that IBS causes.

This study has several strengths. First, to our knowledge, this study will be the first to investigate the effectiveness of EMDR on abdominal pain in IBS in a randomized controlled trial. The association between PTSD and IBS has been well established in various populations [43] and suggestions have been made to expand research into the effect of psychological treatments on IBS symptoms [44]. Two case studies have been published that show that trauma-focused treatment can alleviate IBS symptoms [45, 46], but more systematical and empirical studies are lacking.

Second, we have chosen our various outcome measures to optimize information yield. In the population of IBS sufferers, symptoms tend to vary over time and main symptoms differ per person [9, 20]. To address this problem, we chose to (1) gather data points over an extended period of time (i.e., 2 weeks at baseline, after treatment, and at follow-up) and (2) personalize secondary outcome measures. This provides us with an accurate representation of symptoms and does justice to what is important to the participant. However, to enable comparison to other studies in the IBS realm, several standardized questionnaires are administered that are widely used and recommended [47] in IBS research: the IBS-Severity Scoring System, the IBS-Quality of Life measure, and the IBS-Adequate Relief question. From the IBS-SSS, two measures can be gleaned: (1) the numeric overall score and (2) a dichotomic score of clinically significant improvement of symptoms (50 points reduction of the overall score is considered to represent a clinically relevant improvement) [35].

Third, inclusion in the study will be shortly after being seen by the gastroenterologist. This assures that participants are representative of the patient population referred to secondary care. As the population and conditions reflect clinical practice, generalizability of the results of the study to routine clinical practice is enhanced. Lastly, treatment integrity is ensured by intensive supervision by experts and regular fidelity checks.

There are also some limitations to this study that should be considered. First, our wait-list control condition does not allow us to rule out a positive effect of “therapist contact” on participants’ abdominal pain. Designing an adequate control intervention in research on the effectiveness of psychological treatments is difficult [36, 48]. A proper placebo condition has all components of the experimental treatment, except the actual operating mechanism of this treatment. In psychotherapeutic interventions, all non-specific components of treatment such as time, attention, and contact with therapist should be integrated into the control condition [49]. Moreover, a control intervention which the participants would not consider credible can have a negative impact on the study results and participant motivation causing dropout [48].

Alternatively, comparing EMDR to an active control group, such as gut-directed hypnotherapy or psychoeducation would supply information on the relative efficacy of these treatments.

Due to restrictions in time and resources, we are forced to choose a design that allows us to keep within feasible limits. A proper placebo condition would require more time and resources than we have available. The same applies to the comparison to an active control condition, which would require a larger sample size.

Second, we cannot adjust optimally for a placebo effect. The placebo response in RCTs in IBS is estimated to be considerable [50, 51]. Several recommendations have been made to diminish the effect of placebo response on outcomes of RCTs [49, 52, 53]. We have managed to follow (or nearly follow) the recommendations as to the length of follow-up, the duration of treatment, and using stringent inclusion criteria, but blinding to treatment is impossible, and we have not distinguished between subgroups of IBS (predominant constipation, diarrhea or mixed), or included biomarkers as outcome measures.

Third, it is likely that our sample reflects a selection bias based on the acceptability of the intervention. Medical treatments such as tablets or diet were found to be easily acceptable to most IBS patients, but psychological intervention may not be as readily acceptable to everyone [54]. Both acceptability and credibility of psychological treatment for Persistent Physical Symptoms (formerly Medically Unexplained Symptoms, MUS) are found to be important for successful implementation [55]. It has been suggested that it is crucial that doctors communicate to their patients that attention to psychosocial factors does not preclude vigilance to physical disease. This reduces anxiety about missing a medical problem and enhances the patients’ willingness to accept a biopsychosocial explanation for their symptoms [56]. It may very well enhance the acceptability of psychological treatment as well. In further research, the addition of this assurance by the gastroenterologist may decrease selection bias.

In conclusion, the results of this randomized controlled trial, evaluating the effectiveness of EMDR treatment on abdominal pain in IBS, will contribute to the advancement of IBS management.

## Trial status

Protocol version 2.7 date of approval MEC-U 22–12-2021. Recruitment has started in June 2020 and is still ongoing. The estimated date of completing the recruitment is December 31, 2023.

### Supplementary Information

**Additional file 1. **Participant timeline *Trials* format.

**Additional file 2. ** Informed Consent form

## Data Availability

The research protocol, final trial dataset, and statistical code supporting the findings of the final trial report will be available on request, if deemed reasonable and in accordance with the EU general data protection regulation.

## Abbreviations

AR: Adequate Relief Question
BIG register: Dutch legal register for Professions in Individual Health Care
CCMO: Central Committee on Research Involving Human Subjects in the Netherlands
DSM 5: Diagnostic and Statistical Manual of Mental Disorders, fifth edition
EMDR: Eye movement desensitization and reprocessing
EU: European Union
GLIMMPSE: General Linear Mixed Model Power and Sample Size website
IBS: Irritable bowel syndrome
IBS-SSS: Irritable Bowel Syndrome Severity Scoring System
IBS-QOL: Irritable Bowel Syndrome Quality of Life measure
ID: Identification number
IE: Imaginary Exposure
LEC-5: Life Events Checklist for the DSM 5
LME model: Linear mixed effect model
MEC-U: Medical Research Ethics Committees United
MUS: Medically Unexplained Symptoms
NSAIDs: Non-steroid anti-inflammatory drugs
PCL-5: PTSD Checklist for the DSM 5
PIF: Patient Information Form packet
PPS: Persistent Physical Symptoms
PTSD: Post-traumatic stress disorder
QoL: Quality of life
R: Programming language and software for statistical analysis
RCT: Randomized controlled trial
SUD: Subjective Units of Distress
tCBT: Trauma-focused Cognitive Behavioral Therapy
