# Supplementary material for: The effect of eye movement desensitization and reprocessing (EMDR) on abdominal pain in patients with irritable bowel syndrome (IBS): a study protocol for a randomized controlled trial (EMDR4IBS)
Source: Trials. 2023 Dec 4;24:785. doi: 10.1186/s13063-023-07784-1 (PMC10696837; doi:10.1186/s13063-023-07784-1)
Supplement: Supplementary file 1 — Additional file 1. Participant timeline Trials format. [file 13063_2023_7784_MOESM1_ESM.docx]

|  | STUDY PERIOD | | | | | | | | | | | | | | | | | | | | | | | | |
| --- | --- | --- | --- | --- | --- | --- | --- | --- | --- | --- | --- | --- | --- | --- | --- | --- | --- | --- | --- | --- | --- | --- | --- | --- | --- |
|  | Enrolment | Allocation | Post Allocation | | | | | | | | | | | | | | | | | | | | | | |
| **Timepoint** | pre | 0 | week 1 | week 2 | week 3 | week 4 | week 5 | week 6 | week 7 | week 8 | week 9 | week 10 | week 11 | week 12 | week 13 | week 14 | week 15 | week 16 | week 17 | week 18 | week 19 | week 20 | week 21 | week 22 | post |
| **Enrolment** |  |  |  |  |  |  |  |  |  |  |  |  |  |  |  |  |  |  |  |  |  |  |  |  |  |
| Eligibility screen | X |  |  |  |  |  |  |  |  |  |  |  |  |  |  |  |  |  |  |  |  |  |  |  |  |
| Screen for exclusion criteria | X |  |  |  |  |  |  |  |  |  |  |  |  |  |  |  |  |  |  |  |  |  |  |  |  |
| Informed Consent | X |  |  |  |  |  |  |  |  |  |  |  |  |  |  |  |  |  |  |  |  |  |  |  |  |
| Allocation |  | X |  |  |  |  |  |  |  |  |  |  |  |  |  |  |  |  |  |  |  |  |  |  |  |
| **Inervention** |  |  |  |  |  |  |  |  |  |  |  |  |  |  |  |  |  |  |  |  |  |  |  |  |  |
| Intake* |  |  |  |  | X |  |  |  |  |  |  |  |  |  |  |  |  |  |  |  |  |  |  |  |  |
| EMDR Session 90 minutes* |  |  |  |  |  | X | X | X | X | X |  |  |  |  |  |  |  |  |  |  |  |  |  |  |  |
| **Assessments** |  |  |  |  |  |  |  |  |  |  |  |  |  |  |  |  |  |  |  |  |  |  |  |  |  |
| Diary Questions pain |  |  | X | X |  |  |  |  |  |  | X | X |  |  |  |  |  |  |  |  |  |  | X | X |  |
| Diary Questions complaint 2 |  |  | X | X |  |  |  |  |  |  | X | X |  |  |  |  |  |  |  |  |  |  | X | X |  |
| Diary Questions complaint 3 |  |  | X | X |  |  |  |  |  |  | X | X |  |  |  |  |  |  |  |  |  |  | X | X |  |
| Diary Questions Activity 1 |  |  | X | X |  |  |  |  |  |  | X | X |  |  |  |  |  |  |  |  |  |  | X | X |  |
| Diary Questions Activity 2 |  |  | X | X |  |  |  |  |  |  | X | X |  |  |  |  |  |  |  |  |  |  | X | X |  |
| IBS-SSS** |  |  |  | X |  |  |  |  |  |  |  | X |  |  |  |  |  |  |  |  |  |  |  | X |  |
| IBS-QOL** |  |  |  | X |  |  |  |  |  |  |  | X |  |  |  |  |  |  |  |  |  |  |  | X |  |
| ARQ** |  |  |  |  |  |  |  |  |  |  |  | X |  |  |  |  |  |  |  |  |  |  |  | X |  |
| PCL-5** |  |  |  | X |  |  |  |  |  |  |  | X |  |  |  |  |  |  |  |  |  |  |  | X |  |
| LEC-5** |  |  |  | X |  |  |  |  |  |  |  |  |  |  |  |  |  |  |  |  |  |  |  |  |  |
| **Evaluation** |  |  |  |  |  |  |  |  |  |  |  |  |  |  |  |  |  |  |  |  |  |  |  |  |  |
| Evaluation session |  |  |  |  |  |  |  |  |  |  |  |  |  |  |  |  |  |  |  |  |  |  |  |  | X |
