# Supplementary material for: The effect of eye movement desensitization and reprocessing (EMDR) on abdominal pain in patients with irritable bowel syndrome (IBS): a study protocol for a randomized controlled trial (EMDR4IBS)
Source: Trials. 2023 Dec 4;24:785. doi: 10.1186/s13063-023-07784-1 (PMC10696837; doi:10.1186/s13063-023-07784-1)
Supplement: Supplementary file 2 — Additional file 2. Informed Consent form [file 13063_2023_7784_MOESM2_ESM.docx]

**Informed Consent form**

**The effect of EMDR on abdominal pain in patients with Irritable Bowel Syndrome**

- I have read the participant information letter. I had the opportunity to ask questions. My questions have been answered satisfactorily. I had enough time to decide whether or not to participate.
- I know that participation is voluntary. I also know that I can decide at any time to stop participating in the study. I don't have to give a reason if I do stop.
- I give permission to inform my general practitioner and my gastroenterologist that I am participating in this study.
- I give permission for the collection and use of my data to answer the research question in this study.
- I know that for the purpose of monitoring the study, some people may have access to all my data. These people are listed in the participant information letter. I consent to such access by these persons.
- I give permission for my general practitioner and my gastroenterologist to be informed of any unexpected findings that are or could be important regarding my health.
- I know that I cannot participate in the study if I am pregnant.
- I give permission for video recordings to be made of the treatment sessions.
- I give my permission to be visible in the video recordings of treatment sessions.

**□ yes**

**□ no**

- I give my permission to keep my personal data longer and to use it for future research in the field of EMDR or Irritable Bowel Syndrome.

**□ yes**

**□ no**

- I give my permission to approach me again after this study, and ask me to participate if a follow-up study is started.

**□ yes**

**□ no**

- I want to participate in this study.

Name participant:

Signature: Date : __ / __ / __

----------------------------------------------------------------------------------------------------------------

-

I declare that I have fully informed this participant about the study ***The effect of EMDR on abdominal pain in patients with Irritable Bowel Syndrome***.

If, during the study, any information is found that could alter the participant's consent, I will duly inform them.

Name of investigator (or their representative):

Signature: Date : __ / __ / __

----------------------------------------------------------------------------------------------------------------

-

Additional information has been provided by:

Name:

Position / role:

Signature: Date : __ / __ / __

-----------------------------------------------------------------------------------------------------------------
